# A Protein Deep Sequencing Evaluation of Metastatic Melanoma Tissues

**DOI:** 10.1371/journal.pone.0123661

**Published:** 2015-04-13

**Authors:** Charlotte Welinder, Krzysztof Pawłowski, Yutaka Sugihara, Maria Yakovleva, Göran Jönsson, Christian Ingvar, Lotta Lundgren, Bo Baldetorp, Håkan Olsson, Melinda Rezeli, Bo Jansson, Thomas Laurell, Thomas Fehniger, Balazs Döme, Johan Malm, Elisabet Wieslander, Toshihide Nishimura, György Marko-Varga

**Affiliations:** 1 Oncology and Pathology, Dept. of Clinical Sciences, Lund University, Lund, Sweden; 2 Centre of Excellence in Biological and Medical Mass Spectrometry “CEBMMS”, Biomedical Centre D13, Lund University, Lund, Sweden; 3 Warsaw University of Life Sciences, Warsaw, Poland; 4 National Korányi Institute of Pulmonology, Budapest, Hungary; 5 Clinical Protein Science & Imaging, Biomedical Centre, Dept. of Biomedical Engineering, Lund University, Lund, Sweden; 6 Surgery, Dept. of Clinical Sciences, Lund University, Skåne University Hospital, Lund, Sweden; 7 Skåne University Hospital, Lund, Sweden; 8 Cancer Epidemiology, Dept. of Clinical Sciences, Lund University, Lund, Sweden; 9 Department of Thoracic Surgery, Medical University of Vienna, Vienna, Austria; 10 Section for Clinical Chemistry, Dept. of Laboratory Medicine, Lund University, Skåne University Hospital in Malmö, Malmö, Sweden; 11 First Dept. of Surgery, Tokyo Medical University, Tokyo, Japan; University of Colorado, School of Medicine, UNITED STATES

## Abstract

Malignant melanoma has the highest increase of incidence of malignancies in the western world. In early stages, front line therapy is surgical excision of the primary tumor. Metastatic disease has very limited possibilities for cure. Recently, several protein kinase inhibitors and immune modifiers have shown promising clinical results but drug resistance in metastasized melanoma remains a major problem. The need for routine clinical biomarkers to follow disease progression and treatment efficacy is high. The aim of the present study was to build a protein sequence database in metastatic melanoma, searching for novel, relevant biomarkers. Ten lymph node metastases (South-Swedish Malignant Melanoma Biobank) were subjected to global protein expression analysis using two proteomics approaches (with/without orthogonal fractionation). Fractionation produced higher numbers of protein identifications (4284). Combining both methods, 5326 unique proteins were identified (2641 proteins overlapping). Deep mining proteomics may contribute to the discovery of novel biomarkers for metastatic melanoma, for example dividing the samples into two metastatic melanoma “genomic subtypes”, (“pigmentation” and “high immune”) revealed several proteins showing differential levels of expression. In conclusion, the present study provides an initial version of a metastatic melanoma protein sequence database producing a total of more than 5000 unique protein identifications. The raw data have been deposited to the ProteomeXchange with identifiers PXD001724 and PXD001725.

## Significance

The present study delivers an initial volume of a high-quality mass spectrometry-derived protein sequence database for metastatic melanoma. Complementary benefits of two alternative proteomics approaches are compared. The need for clinically proven biomarkers for application in the diagnosis, staging, and monitoring of treatment of melanoma is critical, which justifies deep mining proteomic analysis of metastatic tissues. Further development and validation of identified proteins aim to deliver markers of clinical utility.

## Introduction

Malignant Melanoma (MM) is defined as cancer of the melanocyte, the cell that produces pigment (melanin) in the skin. Malignant melanoma in a disseminated state has a poor prognosis. According to World Health Organization, there were about 55000 deaths from melanoma occurring globally in 2012 (http://www.iarc.fr/en/publications/books/wcr/index.php).

In Sweden, MM is the sixth most common form of cancer and the one most rapidly increasing with an annual increase around 5% (http://www.Socialstyrelsen.Se/register/halsodataregister/cancerregistret/inenglish).

The majority of early cases of cutaneous melanoma are cured surgically; however some primary tumors will relapse and become metastatic. The American Joint Committee on Cancer staging of the tumors is based on tumor thickness, mitotic rate and ulceration as well as on regional and distant spread [1–3]. Metastatic melanoma has been inherently difficult to treat with a very low 5 year survival (<15%) [4]. Newly developed drugs allowing targeted therapy such as protein kinase inhibitors or drugs modulating the immune response provide more promise [5–10]. However, even with these newer treatments drug resistance may also develop [11]. With treatment options requiring individualized therapies, there is a great demand for validated biomarkers that can support both the primary diagnosis, understanding the progression of disease and response to the treatment of metastatic disease.

Several biochemical markers are already clinically used to monitor progression and relapse of melanoma, such as S100B, MART1 and PMEL [12] and S100A13 [13]. A large number of other markers have been investigated in melanoma, recently reviewed by Levine and Fisher [14]. However, their relevance to melanoma progression, clinical outcome and the selection of best treatment strategies still needs to be established. The search for novel, more accurate markers continues. Both genetic and genomic approaches have been employed in studying MM and specific gene profiles have been correlated to prognosis and survival [15–19]. Gene expression profiles can thus be useful, but identifying and understanding of the functional role of protein in disease development is necessary, for it is proteins rather than genes that are the targets of therapy. Directed protein identification strategies are themselves complex due to the heterogeneities in protein structural components. Proteins can be subjected to a wide variety, even as many as 200, of chemical modifications after translation [20]. These post-translational modifications, often critical to the protein function, may often be altered in disease. Obviously, such modified proteins are also highly important as drug targets.

Various immunological techniques like immunohistochemistry, ELISA, etc. have contributed to build protein expression knowledge and global protein analysis. Technologies such as 2D-PAGE and/or mass spectrometry (MS) have made the identification of an even higher number of proteins possible. A recent publication reports 1528 proteins identified from formalin fixed archival tissue samples of benign nevi, primary melanomas and metastatic melanomas, where 171 proteins differentiated significantly between the three groups [21].

In the present study frozen samples from well-characterized MM tissue, in the South-Swedish Malignant Melanoma Biobank, were utilized [22, 23]. Samples were processed and subjected to chromatographic separation followed by mass spectrometric analysis (LC-MS/MS). The samples were fractionated using strong cation exchange ion chromatography (SCX) in order to increase the number of detected proteins [24]. Protein expression in unfractionated versus fractionated samples was then compared. Data was also analyzed by referring the samples to two melanoma subtype groups based on previously published genomic profiling, where lymph node metastases were grouped by their specific genomic profiles [15, 25]. In that study each “sub-type group” was characterized by differential expression of specific genes; immune-response genes for the “high-immune” group and genes involved in melanin synthesis and melanocyte differentiation for the “pigmentation” group. Also, the sub-type classification was earlier shown to have a prognostic role related to the clinical outcome of the patients [15, 25]

Half of the samples in the current study were obtained from a set defined as the “pigmentation” sub-type and half from samples defined as the “high-immune” subtype [26]. Various statistical techniques were applied to assess biological significance of the protein repertoire detected in the malignant melanoma samples. As detailed in the Methods and Results sections, DAVID analyses related various protein lists to biological processes and pathways, ANOVA analysis was used to look for protein features that might explain why some proteins were, surprisingly, detected only in the unfractionated proteomics approach, and Mann-Whitney U-test was applied to assess differences in protein detection frequencies between sample subsets.

Based on detected protein signatures and molecular classification using bioinformatics analysis, emerging biological relevance could be assigned to several marker proteins.

## Materials and Methods

### Clinical Samples

This study was approved by the Regional Ethical Review Board, Southern Sweden; approval number: DNR 191/2007 and 101/2013. All patients within the study provided a written informed consent. The tumor tissues used were lymph nodes metastases from 10 MM patients undergoing surgery at Lund University Hospital, Sweden. The fresh specimens were divided into two parts. One portion of the metastasis was fixed in formalin, paraffin embedded and sectioned for histopathological confirmation and the other part was snap frozen within minutes after removal and stored at -80°C in the Biobank. The frozen specimens were used as described below for both protein expression analysis and histological comparisons.

### Histology of Tumors

Frozen tissue samples were sectioned on a cryostat into 6 μm thick sections, placed upon glass slides, dried at 37°C for 30 min and fixed with 100% methanol for 5 min. The sections were stained with HE [27, 28], where protein-rich cytoplasm stains dark pink while cytoplasm that is actively synthesizing protein stains rich purple and the nucleus stains blue.

### Sample Preparation

Frozen tissue samples from each tumor were sliced into 10 x 10 μm thick sections using a cryotome. The sections were lysed in 200 μl of 50 mM ammonium bicarbonate and 6 M urea and sonicated with a Branson Sonifier 250 (output 4, 10% duty cycle) for 2 minutes followed by centrifugation at 10 000 g for 5 minutes. The amount of protein in the samples was determined by the BCA method (Pierce, Rockford, IL). A fixed amount (150 μg) of protein were reduced with 10 mM DTT (1 h at 37°C) and alkylated using 40 mM iodoacetamide (30 min, kept dark at room temperature) followed by buffer exchange with 50 mM ammonium bicarbonate buffer (pH 7.6). The samples were then digested with sequencing grade trypsin (Promega, Madison, WI) in a ratio 1:120 w/w (trypsin:protein) overnight at 37°C. The digestion was stopped by adding 30 μL 1% formic acid. The samples were dried using a centrifugal evaporator and resuspended in 150 μL 1% formic acid and centrifuged for 5 min at 10 000 g. The supernatants were stored at -80°C until further use.

### Sample Fractionation

Strong cation exchange chromatography was performed using Microspin columns (MA SEM HIL-SCX, 10–100μg capacity, The Nest group Inc., South Borough) according to the manufacturer’s instruction. The amount of peptides loaded to the columns corresponded to 50μg protein. The peptides were eluted by stepwise salt gradient using 0, 20, 40, 60, 100 and 500 mM KCl in 10 mM potassium phosphate, 20% acetonitrile, pH 2.8. The eluted fractions were dried using a centrifugal evaporator and resuspended in 0.1% TFA in water. Before LC-MS/MS analysis the fractions were desalted using Ultra Microspin column Silica C18 (SUM SS18V, 3–30 μg capacity, The Nest group Inc., South Borough) according to the manufacturer’s instruction. After elution with 50% ACN, 0.1% TFA in water, the fractions were dried using a centrifugal evaporator and each fraction was resuspended in 15 μL 1% formic acid.

### LC-MS/MS Analysis of the Tumor Lysate Digest

The samples were first loaded onto a trapping column (150 mm x 20 μm, Thermo Scientific, San José, CA, USA). The samples were then separated using a column (50 cm x 75 μm, C18, 2 μm and 100Å, PN 164540, Thermo Scientific, San José, CA, USA) with a flow rate of 250 nL/min. A nonlinear gradient was used, using solvent A (0.1% formic acid) and solvent B (0.1% formic acid in ACN). The gradient started with 5% B and 20% B at 120 min, followed by 40% B at 180 min, increased to 90% at 185 min, which was maintained for 5 min.

Both unfractionated and fractionated tumor lysate digests were analyzed using a Q-Exactive, using top10 data-dependent approach. Full MS scans were acquired in the Orbitrap mass analyzer over m/z 350–1800 range with resolution 70,000 (at m/z200). The target value was 3.00E+06. The ten most intense peaks with charge state≥2 were fragmented in the HCD collision cell with normalized collision energy of 30%, and tandem mass spectra were acquired in the Orbitrap mass analyzer with resolution 17,500 at m/z 200. The target value was 1.00E+05. The ion selection threshold was 3.30E+05 counts, and the maximum allowed ion accumulation times were 100 ms for full MS scans and 150 ms for tandem mass spectra. For all the experiments, dynamic exclusion was set to 90 s. The total protein amount of un-fractionated tumor lysate digest injected to the MS/MS platform was 1.25 μg and for the fractionated samples the protein amount was estimated to be 1 μg (50 μg of peptides were fractionated into 6 fractions, approximately 8.3 μg in each fraction). The fractions were dried and resuspended in 15 μL 1% formic acid which gave a concentration of 0.6 μg/μL and 2 μL was injected to the LC-MS/MS).

### Data analysis

Raw data were analyzed with Proteome Discoverer v 1.3 (Thermo Scientific, San José, CA, USA) using both Sequest and Mascot search engines. Uniprot Human (release 06/03/2013) database was used. Precursor and fragment mass tolerances were set to 1 Da, where the maximum of missed cleavage sites was three and 1% false discovery rate was used. At least two unique peptides were necessary for protein identification, and at the parameter Peptide Confidence in the Proteome Discoverer was required to be at least “medium”. The mass spectrometry proteomics data have been deposited to the ProteomeXchange Consortium [29] via the PRIDE partner repository with the dataset identifiers PXD001724 and PDX001725.

### Bioinformatics

Protein lists were analyzed using the DAVID tool set [30]. Enrichment of the lists in particular functional annotations was assessed in DAVID by Fisher's exact test with Benjamini-Hochberg correction for multiple tests. Generally, annotations were considered significant if the Fisher's exact test p-value after Benjamini-Hochberg correction was below 0.001. In this application of the Fisher's exact test, sample sizes were relatively large, specifically; the protein lists analyzed here numbered hundreds of proteins. In a single case, the protein list analyzed by the Fisher's test was small. This was an inherent result of comparison of occurrence frequencies between the melanoma subsets. Gene Ontology annotations, SwissProt keywords, and KEGG pathways were used as annotation terms for the DAVID enrichment analysis. For lists of proteins with significantly changed expression, the background protein sets consisted of all proteins detected by applying the given proteomics approach (fractionated or unfractionated). For the list of all proteins detected, the background protein set consisted of all human proteins. Proportional area Venn diagrams were created using the BioVenn program [31]. Other visualizations, list manipulations and charts were conducted in Spotfire (Somerville, MA, USA, www.tibco.com).

For statistical analysis of significant differences in protein occurrences between the “pigmentation” and “high immune” patient sample sets, Mann-Whitney U test was used as implemented in the in XLSTAT program. For the purpose of this test, for each protein, each sample was assigned an integer value between 0 and 3, corresponding to the number of times the protein was detected in replicates of the sample. Thus, for the unfractionated approach, for each protein the Mann-Whitney test compared two populations of five elements each. Differences in protein occurrences between the patient sample sets were considered significant if the Mann-Whitney U test p-value was below 0.05. In this application of the Mann-Whitney U test, sample sizes were relatively small, namely five patients in each group, as available in this pilot study. For the fractionated approach, sample numbers were too low to attempt a statistical evaluation of differences. This conservative approach to comparison of detection rates was selected to look for strong trends in differences in detection rates between the two sample sets. The ANOVA analysis was performed using the STATISTICA package (StatSoft) with default parameters, and the Least Significant Difference test. Differences in protein properties between sets of proteins detected using the different approaches (see Results) were considered significant if the ANOVA test p-value was below 0.001. In this application of the ANOVA test, sample sizes were relatively large; the groups that were compared numbered hundreds of proteins.

## Results

**Fig 1:**
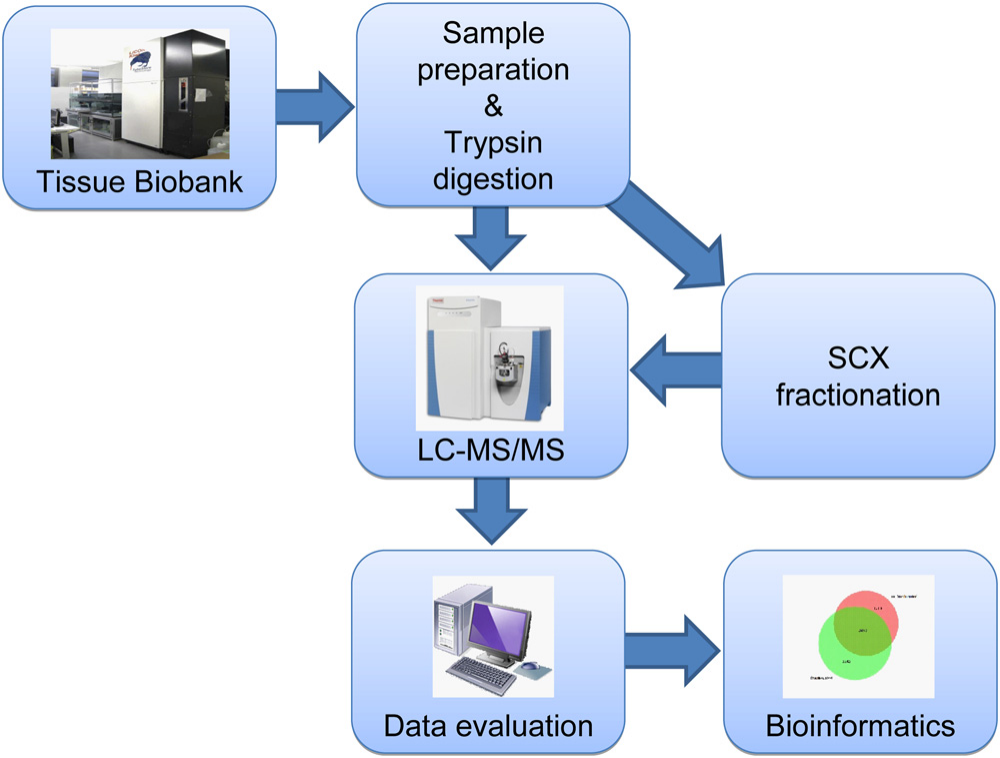
Illustration of study work flow.

The current investigation was performed utilizing a study workflow, as illustrated in Fig 1. The patient characteristics are summarized in Table 1. In the unfractionated approach, ten malignant melanoma metastasis samples were studied, each with three technical replicates. In the fractionated approach, only four out of those ten samples were studied, again each with three technical replicates.

**Table 1:** Patient and tumor characteristics. Breslow tumor thickness and Clarks level of invasion refer to the primary melanoma.

Tumor	Gender	Age at primary melanoma	Years from primary diagnosis to diagnosed metastasis	Breslow class (T class)	Clark	Status
MM35	Male	54	1	3	4	Alive
MM98	Male	73	2	4	4	Dead
MM504	Male	NA				Dead
MM687	Male	72	2	1	2	Dead
MM787	Male	78	78	2	4	Dead
MM812	Male	NA				Alive
MM813	Female	54	0	2	3	Alive
MM825	Female	64	2	2	4	Alive
MM829	Male	49	6	1	2	Alive
MM835	Female	32	4	3	3	Alive

*NA—not available, primary tumor not diagnosed

### Histological Characterization

**Fig 2:**
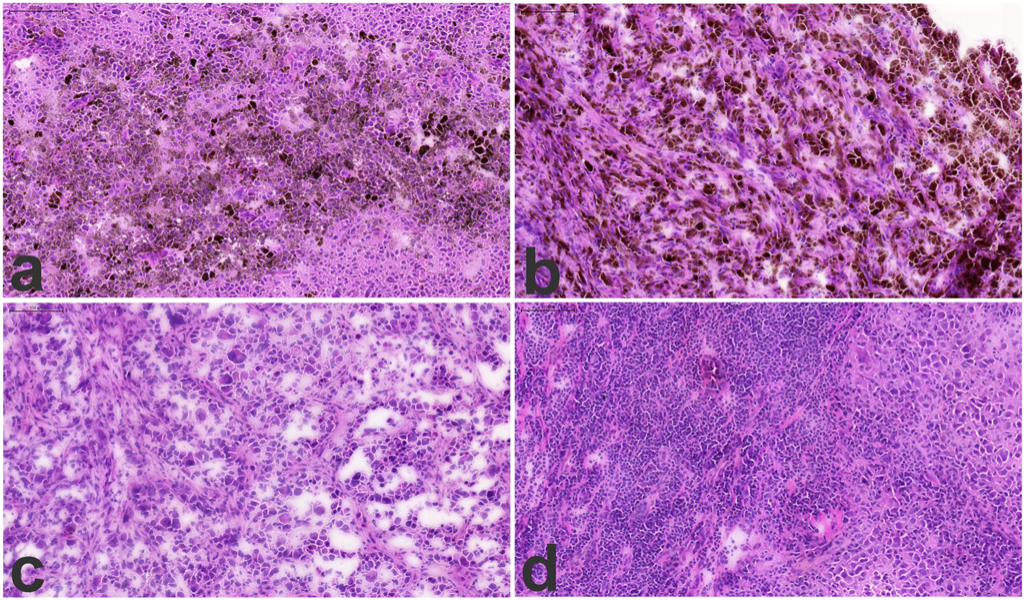
Histology images of lymph node metastasis, 2a and b the “pigmentation” subtype and 2c and d the “high-immune” subtype. Frozen tumor samples were cryosectioned and stained with HE. The nuclei stains blue and protein-rich cytoplasma stains dark pink while cytoplasma that is actively synthesizing proteins stains rich purple. The brown pigment seen scattered within clusters in 2a and b corresponds to focal hyper-expression of melanin by groups of melanoma cells.

Characterization of the tissue samples by histology of the lymph node tumors showed almost complete replacement of normal follicular architecture by metastatic melanoma cells, as shown in Fig 2 (2a and b represent the “pigmentation” subtype and 2c and d represent the “high-immune” subtype).

### Deep mining—Protein Sequencing

#### Proteins detected using the two proteomics approaches

Since, rather unexpectedly, some proteins were only identified in the samples run without the SCX step (unfractionated) we decided to include a protein expression comparison that relates the peptide and protein annotations between unfractionated, and orthogonal- fractionated separation. The rationale here was to look for any factors that might contribute to the unexpected gain in protein identifications by combining the two proteomics approaches. Thus, the output of protein identifications can be related to the cycle time needed for a given work flow procedure. The most common bottom-up LC-MS platform used today in clinical protein expression studies is C18-hydrophobic separation. This methodology provides high resolution liquid phase nano-capillary separation of peptide sequences, where typically 1–4 hour organic solvent gradients are being applied. In order to increase the resolving power, an ion-exchange separation step can be introduced either on-line, or off-line as done in the present study [32–34].

The electrostatic charge properties will provide additional beneficial factors in the overall work flow for the separation efficiency in a proteomic study [35, 36].

The resulting outcome was evaluated and analyzed with pre-set criteria for protein sequence annotation confirmation (see Materials and Methods).

**Fig 3:**
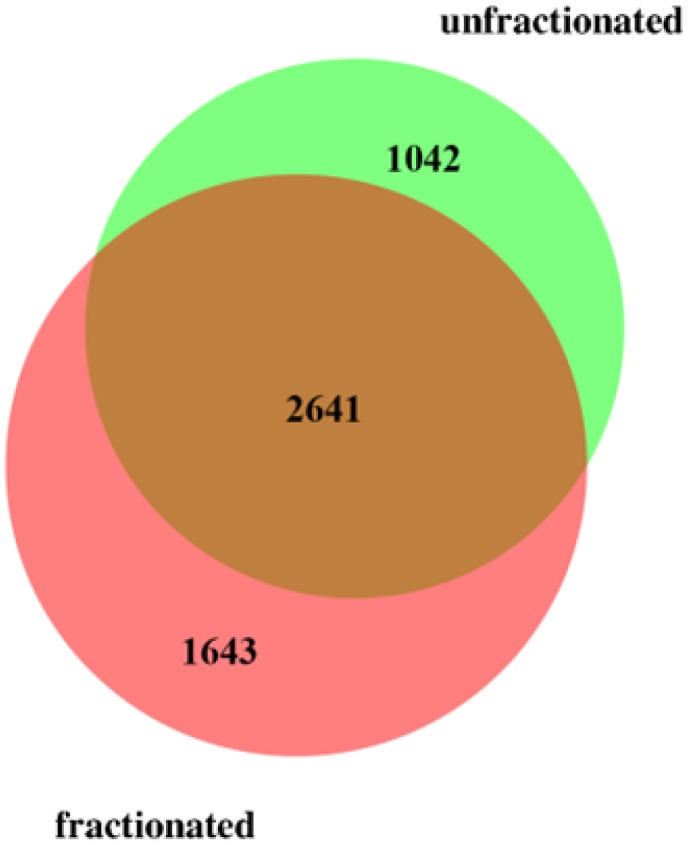
Venn diagram showing overlap between proteins detected applying the fractionated and the unfractionated approaches.

The total number of annotated proteins detected was 5326 using the conservative statistical criteria used (see Materials and Methods), combining both the fractionated and unfractionated approaches, and using the more than 17000 unique peptides identified. Among these, 4284 proteins were annotated using more than 13600 peptides by applying the fractionated approach, while without fractionation 3683 proteins were annotated using more than 14700 peptides. Even though it is well known that pre-fractionation is favorable for analysis in order to maximize proteome coverage, our study showed that more than 1000 detected protein were only found using the unfractionated approach. The overlap between the two approaches was 2641 proteins, i.e. close to half of the total number of proteins detected (Fig 3 and S1 Table).

A substantial proportion of proteins were detected with few peptides only, e.g. in the unfractionated approach 2360 proteins (61% of all proteins detected) and in the fractionated approach 2375 proteins (58%) were detected with 2 or 3 peptides. Also, a substantial proportion of proteins were detected with relatively low sequence coverage, e.g. 1631 proteins (42%) had coverage below 10% in the unfractionated approach while 2169 (53%) had coverage below 10% in the fractionated approach. A substantial proportion of proteins were detected only in one or two sample, e.g. as many as 1443 proteins (37% of all proteins detected) were detected in 1 or 2 samples in the unfractionated approach. In the fractionated approach 2311 proteins (56%) were detected in 1 or 2 samples only. Proteins detected in almost every sample (all samples or all but one) were relatively few, 585 and 245 in the fractionated and unfractionated approaches, respectively.

#### Comparison between the fractionated and unfractionated approaches to proteomics

The proteins specific (unique) to each of the two proteomics approaches exhibited rather few special functional characteristics. Here, the DAVID tool set was used to look for biological processes and pathways that may be linked to or characteristic for the two protein sets. Thus, DAVID analyses of overrepresented functional annotations yielded following Gene Ontology (GO) annotations: MHC complex, mitochondrial outer membrane and DNA binding for the unfractionated approach-unique protein set. For the fractionated approach-unique proteins, Pleckstrin homology domains, and Gene Ontology (GO) terms such as: GTPase regulator activity, zinc-finger, protein kinase activity were significantly over-represented.

Another DAVID analysis has been performed to look for biological processes and pathways that may be linked to or characteristic for the full set of proteins detected in the current experiment (combining the unfractionated and fractionated approaches). Thus, the full set when compared with human proteome in general was characterized by overrepresented functional annotations that could be expected for melanoma samples. Specifically, GO: “melanosome”, GO:“pigment granule”, GO:“cytoplasmic vesicle” annotations were found with p-values below 1E-15, and may correspond to melanocyte-specific protein groups. Then, the following terms may correspond to regulation of cancer-specific processes: KEGG: “Glycolysis”, p-value approx. 1E-8, GO: Mitochondrion, p-value 1E-37, GO: RNA binding and RNA splicing, p-values below 1E-25.

**Fig 4:**
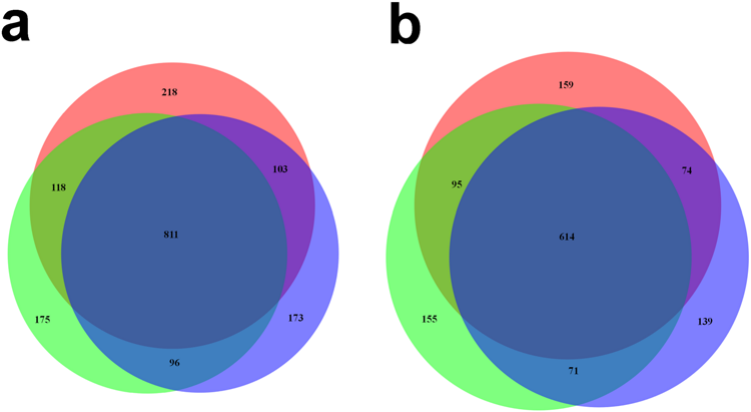
Venn diagram for unfractionated samples. Proteins seen in all three replicates *vs* those seen in a single replicate, for two typical patient samples (a and b).

Interestingly, separate analysis of technical replicates provided a substantial gain in number of proteins detected. As seen in Venn diagrams for two typical unfractionated patient samples (Fig 4a and 4b), approximately half of proteins detected in a sample were observed in all the three replicates while approximately a third of the proteins are observed in a single replicate only.

There is a major impact on n-numbers of identified peptides and resulting proteins, depending on the work flow chosen in melanoma patient tissues, in addition to the uniqueness of the protein sequences. The fractionated workflow provides an increase in number of detected proteins in relation to the unfractionated approach. One explanation of why we also find different proteins in unfractionated and fractionated LC-MS workflows is that hydrophobic separation mechanisms are being utilized for separation in unfractionated samples, while we have an electrostatic mechanism prior to the hydrophobic separation in the fractionated workflow. This means that the fractions being introduced into the 2^nd^ dimension of separation, already have been fractionated based on the respective peptide polarity, given at the specific pH, and the increasing salt concentrations. These orthogonal properties will be different in combination (charge and hydrophobicity), in relation to not using fractionation, where only the apolar properties will impact the separation.

When physical properties of proteins detected a) by only using the fractionated approach, b) by only using the unfractionated approach and c) by using both approaches, were compared by the ANOVA technique, interesting patterns emerged. All three conditions were related to significantly different average molecular weight (79 kDa, 56 kDa and 68 kDa, respectively, p-value<0.004 for any of the three contrasts). As expected, similar differences were observed for the average lengths of the proteins detected. Thus, the fractionated approach-unique proteins were significantly larger, on average, than the others.

Among detection parameters, the average sequence coverage for proteins detected: a) using only the fractionated approach, b) using only the unfractionated approach and c) using both approaches, was also significantly different between the conditions (8.9%, 12.2% and 15.6%, respectively, p-value <0.0001 for any of the three contrasts).

Other detection parameters exhibited different variation patterns. Number of unique peptides per protein or number of peptide-spectrum matches (PSMs was significantly higher for proteins detected using both approaches (ANOVA p-value <0.0001), with borderline or no difference between proteins detected using solely one of the approaches.

This fact may suggest that proteins detected exclusively using a single approach (either without fractionation or with fractionation) are lower in abundance, although rigorous quantification was not performed here.

#### Comparison between “pigmentation” and “high-immune” subtypes of melanoma

**Fig 5:**
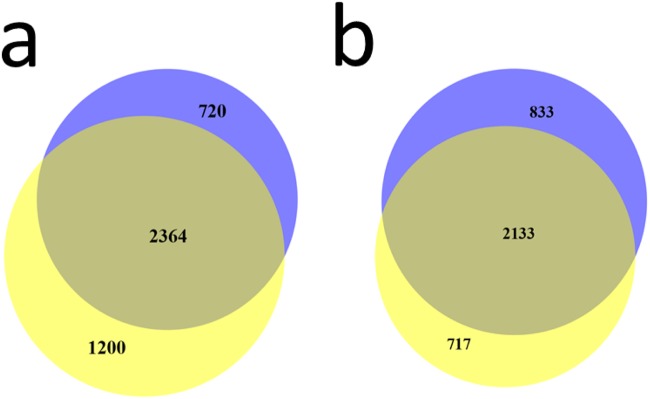
Venn diagrams for a) fractionated approach and b) unfractionated approach. Proteins seen in the “pigmentation” (blue color) sample subset *vs* those seen in the “high-immune” (yellow color) sample subset.

Both proteomics approaches allowed comparison between two subtypes of melanoma identified previously in a genomics study, the “pigmentation” and the “high immune” subsets [15, 37]. Substantial differences could be observed in protein presence (detection) between the two subsets, with only about 50% of the proteins detected in both subsets (Fig 5a and 5b shows these differences for fractionated and unfractionated approaches, respectively).

**Fig 6:**
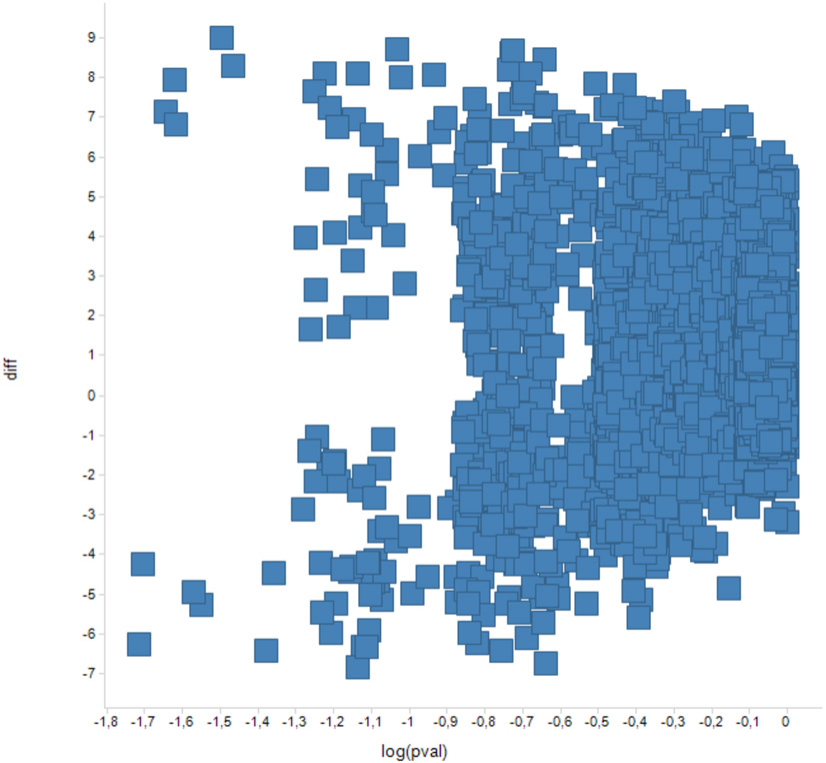
Scatter graph—Y axis: protein detection counts in the “pigmentation” subset minus protein detection counts in the “high-immune” subset. X axis: Mann Whitney test p-values for the differences.

However, more biologically relevant would be comparison of protein detection frequencies between the subsets. Due to the small numbers of patient samples in this pilot study, protein presence (detection) frequency could only be compared between the “pigmentation” and the “high-immune” melanomas for the unfractionated approach. This was done using Mann-Whitney U test for comparison of 5 versus 5 samples. For every sample, the detection frequency could assume values of 0, 1/3, 2/3 and 1. With the threshold for the p-value of the Mann-Whitney U test set at 0.05, there were 11 proteins differing in detection frequencies between the “pigmentation” and the “high-immune” subsets in the unfractionated approach (Fig 6 and Table 2).

**Table 2:** Proteins differing in detection frequency between the “high-immune” and the “pigmentation” melanoma sample subsets. Detection counts include replicates.

Protein ID	Mann Whitney—p-value	Count in “pigmentation” subset	Count in “high-immune” subset	Description
P47756	0,019	8	0	F-actin-capping protein subunit beta (CapZ beta); CAPZB_HUMAN
Q9UJU6	0,019	14	5	Drebrin-like protein (Cervical SH3P7); DBNL_HUMAN
B7Z1I0	0,023	0	4	Integrin-linked protein kinase; ILK_HUMAN
E5RIW3	0,024	0	4	Tubulin-specific chaperone A; E5RIW3_HUMAN
P04264	0,024	0	7	Keratin, type II cytoskeletal 1 (67 kDa cytokeratin); K2C1_HUMAN
Q99733	0,025	15	7	Nucleosome assembly protein 1-like 4 (Nucleosome assembly protein 2); NP1L4_HUMAN
Q9NZM1	0,025	0	6	Myoferlin (Fer-1-like protein 3); MYOF_HUMAN
O95881	0,042	12	4	Thioredoxin domain-containing protein 12 (Endoplasmic reticulum resident protein 18); TXD12_HUMAN
Q8IZP2	0,042	14	5	Putative protein FAM10A4 (Suppression of tumorigenicity 13 pseudogene 4); ST134_HUMAN
Q9UH99	0,043	1	8	SUN domain-containing protein 2 (Protein unc-84 homolog B); SUN2_HUMAN
P31930	0,049	5	9	Cytochrome b-c1 complex subunit 1, mitochondrial; QCR1_HUMAN

The rigorous Mann Whitney test yielded a small number of proteins differing in detection frequency between the “high-immune” and “pigmentation” sets. Hence, analysis of overrepresented functional terms had low statistical power. Here, DAVID analysis has been performed to look for biological processes and pathways that may be linked to or characteristic for the proteins differing in detection frequency between the “high-immune” and “pigmentation” sample sets. The only term reaching significance in the DAVID analysis even without multiple test correction was “cellular stress response”. This suggests that the differences between “high immune” and “pigmentation” sample sets may be related to these processes. One protein recently described to be expressed in melanoma is alpha-synuclein [38, 39]. Dysregulation of alpha-synuclein is observed in Parkinson’s disease and the protein is implicated in the pathway of dopamine as well as melanin synthesis. In the present study, the differential expression of alpha-synuclein between the “pigmentation” and “high-immune” melanomas did not reach statistical significance, however a trend was clear. Thus, alpha-synuclein was observed in 4 “pigmentation” tissue samples and 3 “high-immune” tissue samples using the unfractionated approach, and 2 “pigmentation” samples and 1 “high-immune” using the fractionated approach.

This trend is interesting remembering relatively poor prognosis of patients with pigmentation subtype of melanoma [15]. In our earlier study we found a strong correlation between protein and mRNA expression levels of alpha-synuclein in metastatic tumor lysate from MM [39].

A recent study by Byrum et al. utilized Formalin-Fixed, Paraffin—Embedded tissues samples and compared protein abundances between benign, primary and metastatic melanomas [21]. These melanoma categories do not translate directly into “pigmentation” and “high-immune” melanoma subsets. However, out of 171 proteins that they reported to be differentially expressed between the conditions studied, 147 proteins were detected in our study. The vast majority of the significantly differentiating proteins reported by Byrum et al. were detected in their metastatic melanoma samples, and approximately half of these were actually upregulated in metastatic melanoma vs primary or malignant samples.

Another recent study by Mactier and co-workers elucidated protein signatures related to survival outcome in melanoma patients with stage IIIc lymph node metastases [40]. Out of 84 proteins they reported to be differentially abundant between the prognostic groups, 76 proteins were detected in our study. Thus, for both studies, the majority of proteins differentiating between the respective clinically relevant conditions are detected in our study.

Among a number of melanoma markers being considered in the literature (https://www.aacc.org/SiteCollectionDocuments/NACB/LMPG/tumor/chp3L_melanoma.pdf Table 2b Tissue tumor markers), five proteins exhibited substantial expression in our experiment (presence in at least 40% of the samples in either approach): S100B, ICAM, NDKA (NM23), MUC18 (MCAM), PMEL (GP100). Further five proteins were seen in fewer samples (Table 3). Interestingly, MMP9 was seen in 50% of the fractionated samples while it was never seen in the unfractionated ones. Also, both AP-2 and MITF were only detected in a single fractionated sample.

**Table 3:** Present rates for selected melanoma markers proposed in the literature. Percentages of samples (including technical replicates) in which a protein was detected.

Protein	Unfractionated approach	Fractionated approach
S100B	70%	100%
ICAM	43,3%	100%
NDKA (NM23)	46,7%	83.3%
MUC18 (MCAM)	40%	100%
PMEL (GP100)	46,7%	58.3%
MMP-9	0%	50%
CD44	3,3%	25%
Tyrosinase	3,3%	16.7%
AP-2	0%	8.3%
MITF	0%	8.3%

## Discussion

In this study, applying conservative criteria, more than 5000 proteins were identified in metastatic melanoma samples at a 1% false discovery rate. Two approaches of sample preparation using either unfractionated whole tissue lysates or orthogonal fractionation were applied to test the conditions required for optimal MS proteomics annotations. Both the approaches were found to be complementary and each provided identifications of approximately half of the detected proteins. Deep mining the protein sequencing by nano-LC MS, has for over a decade been a challenging task, and still is.

Even in comparison, while studying the individual MS spectra derived from replicates of the same sample and analyzed on the same instrument platform, one has to keep in mind that the current nano-LC MS platforms provide an output that is to some extent variable. The probable factors contributing to these variances in detection precision are already known and include 1) the integral complexity of the protein composition within whole tissue extracts, 2) the power of the nano-chromatographic separation to provide single peptide separation, and rather the separation of multiple peptides that are interfaced into the same nano-ESI interface separation time space, 3) since the mass spectrometer acts as both a separator, as well as a detector, it is unable to generate MS and MS/MS sequence data on all individual peptides present in the sample [41].

The full set of proteins detected herein in metastatic melanoma samples was enriched in functional annotations well expected for melanocyte-containing tissues and in annotations related to regulation of cancer-specific processes. Interestingly, not only alternative proteomics approaches, but also technical replicates provided a very substantial gain in number of proteins detected.

The two protein sets specific (unique) to the two alternative proteomics approaches were enriched in some particular protein groups, e.g. MHC complex and mitochondrial outer membrane proteins (unfractionated approach) or zinc-finger proteins and protein kinases (fractionated approach).

The unique proteins identified by the fractionated approach were significantly larger in size, on the average, than the others. Reasons for this are not clear to us. Probably as a consequence, the average sequence coverage for proteins detected using only the fractionated approach was significantly lower than the coverage for proteins detected using both approaches and those unique to the unfractionated approach. Interestingly, no differences in protein pI were observed between the two approaches. Overall, significantly higher number of unique peptides per protein and numbers of PSMs observed for proteins detected using both approaches when compared to approach-specific proteins may suggest that proteins detected using only a single approach (be it unfractionated or fractionated) are of lower abundance.

A comparison of protein detection frequencies was conducted between the biologically relevant “high-immune” and “pigmentation” sample sets. The few proteins significantly differing in detection frequencies were enriched in cellular stress response annotations.

In addition, our study indicates that a number of possible melanoma markers being considered in the literature can be readily detected in a substantial fraction of our metastatic melanoma samples, even if the experiment was not directed at quantifying the particular proteins.

## Conclusion

We present here the initial stage of a protein sequence database for metastatic melanoma using deep mining high-quality mass spectrometry data. More than 5000 proteins were identified in metastatic tissues using our approach.

Further development of the metastatic melanoma proteomics database will involve larger sample numbers, collection of additional clinical parameters for the samples, development of validation assays (e.g. SRM) directed at particular protein molecules. Larger sample numbers will allow a deeper insight into the biological mechanisms related to metastatic melanoma development and potential differences between disease subtypes.

Increased knowledge about protein expression and regulation during melanoma disease progression and treatment will support development of validated biomarkers for melanoma patients.

## Supporting Information

S1 Table Protein identified using the two proteomics approaches. Protein identified using Uniprot.
(XLS)
